# Aggregation behaviour of amphiphilic cyclodextrins: the nucleation stage by atomistic molecular dynamics simulations

**DOI:** 10.3762/bjoc.11.267

**Published:** 2015-12-07

**Authors:** Giuseppina Raffaini, Antonino Mazzaglia, Fabio Ganazzoli

**Affiliations:** 1 Dipartimento di Chimica, Materiali e Ingegneria Chimica ‘G. Natta’, Politecnico di Milano, via L. Mancinelli 7, 20131 Milano, Italy; 2 Unità Politecnico, INSTM, piazza Leonardo da Vinci 32, 20133 Milano, Italy; 3 CNR-ISMN Istituto per lo Studio dei Materiali Nanostrutturati, c/o Dipartimento di Scienze Chimiche, Biologiche, Farmaceutiche ed Ambientali dell’Università di Messina, Via F. Stagno d'Alcontres 31, 98166 Messina, Italy

**Keywords:** aggregation, amphiphilic cyclodextrins, micelles, molecular dynamics simulations, nanoparticles, self-assembly

## Abstract

Amphiphilically modified cyclodextrins may form various supramolecular aggregates. Here we report a theoretical study of the aggregation of a few amphiphilic cyclodextrins carrying hydrophobic thioalkyl groups and hydrophilic ethylene glycol moieties at opposite rims, focusing on the initial nucleation stage in an apolar solvent and in water. The study is based on atomistic molecular dynamics methods with a “bottom up” approach that can provide important information about the initial aggregates of few molecules. The focus is on the interaction pattern of amphiphilic cyclodextrin (aCD), which may interact by mutual inclusion of the substituent groups in the hydrophobic cavity of neighbouring molecules or by dispersion interactions at their lateral surface. We suggest that these aggregates can also form the nucleation stage of larger systems as well as the building blocks of micelles, vesicle, membranes, or generally nanoparticles thus opening new perspectives in the design of aggregates correlating their structures with the pharmaceutical properties.

## Introduction

**Scheme 1:**
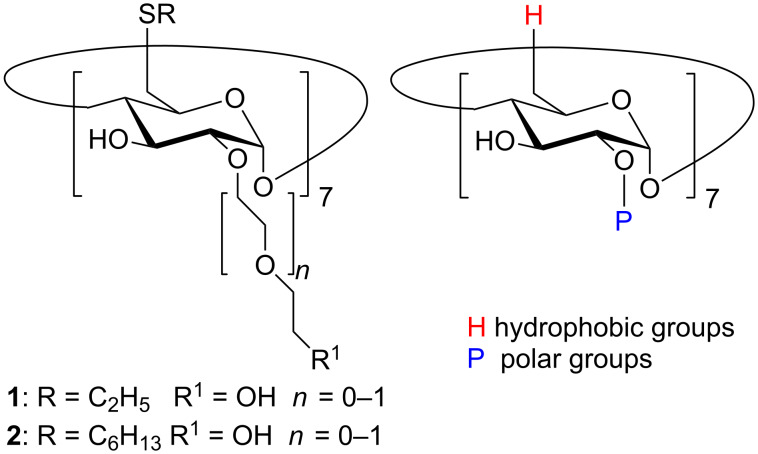
Structure of an aCD functionalized with hydrophobic thioalkyl C2 (R = C_2_H_5_) or C6 (R = C_6_H_13_) chains at the primary rim (hydrophobic H groups) and polar oligoethylene glycol (–OCH_2_CH_2_–)*_n_* chains at the secondary rim (polar P groups).

Inclusion complexes with supramolecular structures formed by native or modified cyclodextrins (CDs) are attracting an increasing attention [1–8], including also the new polymeric CD nanogels [9] and nanosponges [10–13]. Over the past twenty years, amphiphilic cyclodextrins (aCD) formed with α-, β-, or γ-CD have given rise to a wide interest in the scientific community because of their versatility both as drug carriers [11,14–15] and as self-assembling systems for molecular recognition [16–18]. Different research groups investigated the aCD behaviour in solution, elucidating their nanostructures and physicochemical behaviour, including the temperature- and concentration-dependence of the supramolecular structures, or the pH dependence of water solubility, so as to improve our understanding of their activity as drug delivery systems [19–20] and of the biological fate of the assemblies [21–22]. The balance between the hydrophobic and the polar groups on the two CD rims modulates the formation of micelles, vesicles, nanospheres (or dense aggregates), and nanocapsules [1]. In particular, non-ionic aCD obtained from β-CD modified with hydrophobic thioalkyl chains (H groups in the following) at the primary rim and short polar PEG oligomers (P groups) at the secondary rim form micelles and micellar clusters that are increasingly dispersible in water when functionalized with thioalkyl C2 or C6 chains [23–24] (see Scheme 1), or vesicles with C12 or C16 chains, respectively [17,22].

Potential applications of non-ionic aCD as anticancer and antiviral drug nanocarriers were recently reported [14], while analogue cationic aCD with terminal short amino-PEG at the secondary rim form nanoassemblies which entrap photosensitizers for photoactivated therapy [25] or DNA for gene delivery [26–30]. The potential of aCD is strengthened by their ability to selectively recognize cells by exposing receptor-targeting groups on the surface of the nanoassembly [30]. Because of these promising results, we have begun to investigate the aggregation behaviour of an aCD model compound by atomistic computer simulation to clarify the early stages of self-assembly, in particular the aCD interactions in the nucleation stage, and give insights on the structure of the embryonic building blocks of the aCD’s supramolecular nanosystems. We also note that in the case of a kinetic control of aggregation taking place by sequential interaction of further aCD, the nature of these embryonic building blocks may affect the structure and stability of the larger aggregates.

Some papers already reported simulation studies of CD aggregates, or better dimers, in water in the presence of hydrophobic or at least amphiphilic moieties, such as ionic [31] and non-ionic [32–34] surfactants assuming a preassembled state with the hydrophobic chains threading through one or two native CDs (see also the non-covalent super-amphiphilic complexes described in [33–34]), or of the unbiased aggregation process of two larger CDs encapsulating C_60_ [35]. Other studies considered again preassembled micelles, such as for instance the wormlike micelles formed by the cetyltrimethylammonium cations, investigated at various salt concentrations to assess their stability against rupture in smaller spherical micelles [36], or more recently a bilayer of aCDs functionalized through an anthraquinone moiety mimicking a small portion of a whole vesicle [37]. Otherwise, coarse-grained Monte Carlo simulations in two dimensions modelled the self-assembly of aCD [38]. It should be underlined, however, that in the atomistic simulations a manually pre-assembled system was generally assumed, while the spontaneous formation of supramolecular aggregates was seldom, if ever, considered, apart from the above-mentioned reference [35]. To improve our understanding of the factors driving the formation of aCD molecular assemblies, we describe in this paper an atomistic molecular dynamics investigation of a model compound of a non-ionic aCD extensively studied experimentally [23–24]. The aim of the present work is to describe the first aggregation step that eventually leads to formation of a micelle or more generally of a large aggregate that may be held together through the interaction both within the cavity and, at the outer surface, by a combination of dispersion and dipolar interactions and of hydrogen bonds, adopting throughout a “bottom up” atomistic description.

The modelled system consists of an amphiphilic β-CD of Scheme 1 carrying hydrophobic H groups at the primary rim (R = C_2_H_5_) and polar P groups at the secondary rim with *n* = 0 (R^1^ = OH), simply denoted in the following as the model aCD. The simulations used molecular mechanics (MM) and molecular dynamics (MD) methods, and were carried out both in vacuo, to mimic a non-polar and weakly interacting solvent, and in explicit water, using a box of water molecules with periodic boundary conditions (PBC). While MM methods involve energy minimizations of the simulated systems with respect to all the atomic coordinates, the MD methods describe the time evolution of the whole system at the chosen temperature, according to Newton’s equation of motion, thus following the kinetics of a process and the system equilibration, within the accessible simulation time. As previously done [35,39–42], we employ a standard simulation protocol subsequently adopted also by other groups [43]: First we carry out an initial energy minimization of trial geometries mimicking a random approach of the molecules in solution, and then we perform MD runs of these geometries until equilibrium, monitored inter alia through the system energy and its components, and through the intermolecular separations, is achieved. Eventually, we carry out final optimizations of different conformations saved during the MD runs after equilibration to determine the interaction energy and the system geometry, either in the most stable final state or in some largely populated geometry met within the dynamic run in order to characterize the main features of the (pseudo) equilibrium nucleation states.

In the following, after the methodological section, we first discuss the conformation of the isolated molecule of the model compound to determine the intramolecular conformation in vacuo and in water. We then model the interaction between two molecules in vacuo and in water considering three different mutual orientations variously facing the H and P groups to have information about the stability of the contacts among the hydrophobic and/or the polar substituents. Afterwards, we study more briefly the interaction among four molecules, mentioning also some preliminary results of larger systems. The final section summarizes the main results with an outlook to future work.

## Simulation Method

The simulations were performed with InsightII/Discover 2000 [44], using the consistent valence force field CVFF [45] as previously done [35,39–40,46]. The geometry of the model aCD, generated with the available templates of InsightII, was subjected to an MD run in vacuo and in explicit water at 300 K, and finally optimized up to an energy gradient lower than 4 × 10^−3^ kJ mol^−1^ Å^−1^. The aggregate formation was modelled by placing the appropriate number of molecules in different trial arrangements (see later), so that the different CD rims could face one another. The hydrated systems were modelled after adding a large number of water molecules at the local density of 1 g cm^−3^ in prismatic cells of appropriate size, adopting periodic boundary conditions (constant-volume conditions). These molecules were then modelled exactly in the same way as the solute molecules. After an initial geometry optimization, the resulting adducts were subjected to independent MD runs and final geometry optimizations considering in vacuo many different geometries saved during the MD run, and in water the final configuration at equilibrium (the simulation length, dependent on the system size, will be mentioned in the text). The dynamic equations were integrated using the Verlet algorithm [47] with a time step of 1 fs at a temperature of 300 K, controlled through the Berendsen thermostat [48], and the instantaneous coordinates were periodically saved for further analysis. The system equilibration was monitored by the time change of the total and potential energy of the system and of its components, and of relevant intermolecular distances, in particular those between the centres of mass of the interacting macrocycles. Based on these equilibration criteria, the MD runs were carried out for different lengths. The simulations in explicit water were often shorter than in vacuo due to the much larger computational burden of a fully hydrated system, so that much lengthier rearrangements cannot be ruled out. On the other hand, system thermalization is significantly faster in water than in vacuo due to the random collisions with the solvent molecules, which compensates in part the difference in the length of the MD runs.

The geometries periodically sampled in the MD runs were analysed through the pair distribution function *g_ij_*(*r*), or PDF, as described for instance in [49]. This function gives the probability density of finding atoms *j* at a distance *r* from atoms *i*, and is defined here in the non-normalized form as

[1]
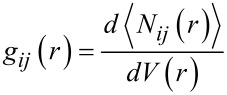


where 
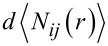
 is the average number of times the *j* atoms are comprised in a spherical shell of thickness *dr* at a distance *r* from atoms *i* within an MD run. Thus, *g_ij_*(*r*) yields the average non-normalized probability of finding of atoms *j* in the shell volume *dV*(*r*) at a distance between *r* and *r* + d*r* from atoms *i*, giving an immediate picture of the local density of *j* atoms due to specific interactions.

## Results and Discussion

### The isolated molecule in vacuo and in explicit water

**Figure 1:**
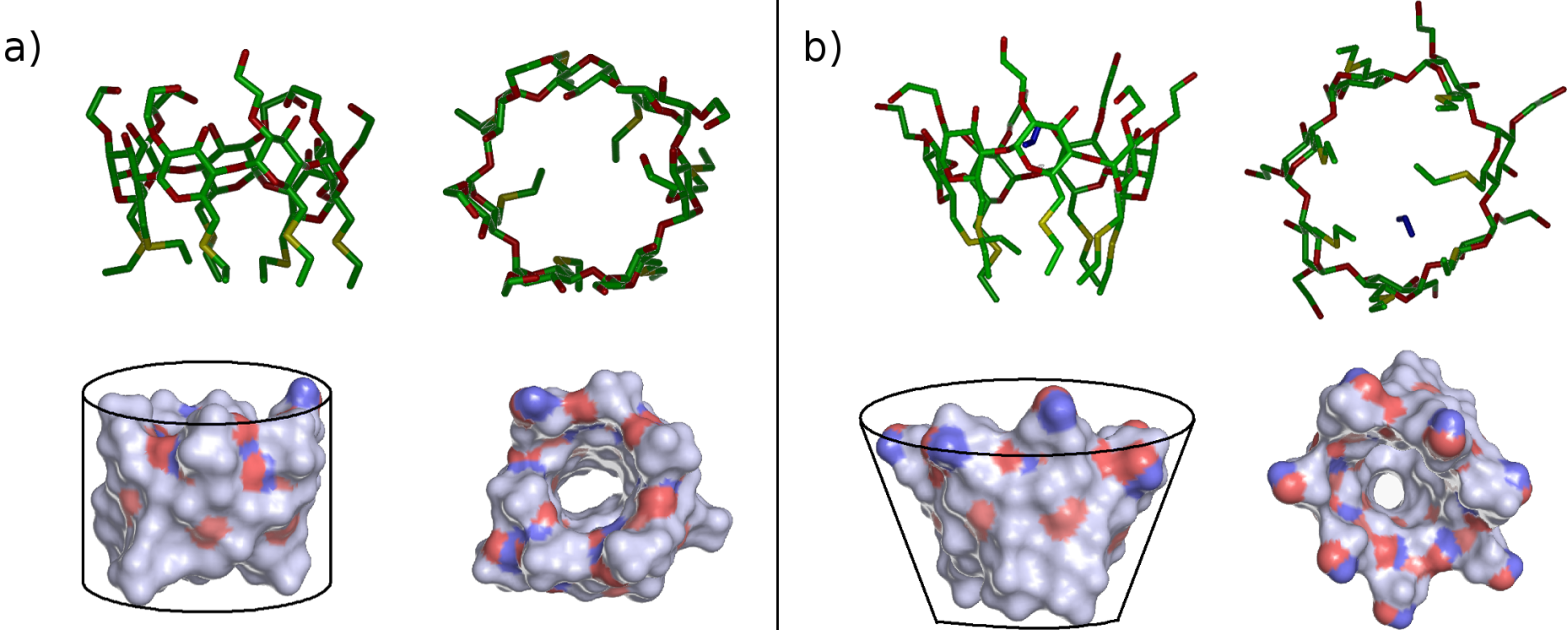
The final optimized geometry of the aCD molecule in vacuo (panel a) and in explicit water (panel b) viewed sideways (at left) and from above the polar groups (at right). In each panel, the upper part shows the line drawing of the molecule (carbons are in green, oxygens in red, and the sulfur atoms of the H chain in yellow, while the hydrogens were omitted for clarity) and the lower part the surface accessible to the solvent, considered as a spherical probe with the radius of 1.4 Å (the electrically neutral part of the molecule is in grey, the negative areas of the oxygens are in red and the positive ones of the hydroxy hydrogens in blue). In part b, we also show a water molecule entering the cavity to replace another one present at the beginning of the MD run (see text).

**Figure 2:**
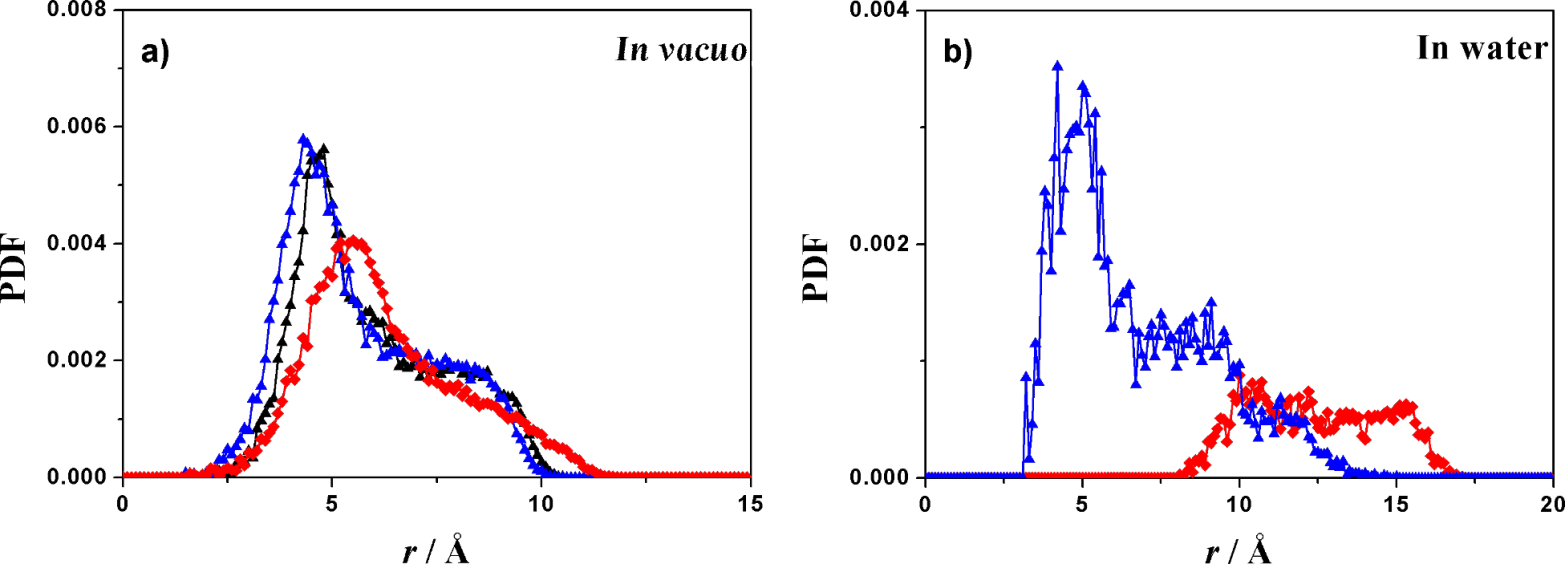
Some relevant PDF’s calculated along the runs for the isolated aCD. a) The PDF of the glycosidic oxygens (black and blue symbols) of the polar secondary rim and of the S atoms (red symbols) of the hydrophobic primary rim as a function of their separation from the macrocycle c.o.m. in vacuo. b) The PDF of the glycosidic oxygens carrying the P chains (red symbols), and of the S atoms carrying the H chains (blue symbols) as a function of their separation from the macrocycle c.o.m in water.

Using the above-mentioned simulation protocol proposed by some of us [39–42], we first studied the isolated aCD molecule. After the initial minimization, the MD run at room temperature lasting for 5 ns, and the final optimizations of 200 snapshots saved along the trajectory, we obtained the most stable geometry in vacuo. The simulations show a weak clustering of the hydrophobic thioalkyl groups and an extensive pattern of hydrogen bonds at the polar rim involving the adjacent OH groups (Figure 1a), yielding a cylindrical molecular shape with similar diameters of the two rims. This shape is qualitatively displayed by the internal molecular cavity shown in Figure 1a, and quantitatively revealed by the similarity of the pair distribution function PDF of the glycosidic oxygens on the macrocycle and of the S atoms carrying the H chain as a function of their distance from the macrocycle centre of mass (c.o.m.), shown in Figure 2a. In particular, these distances roughly fluctuate around a similar average value, with a similar shoulder at larger separation. We further note for the later discussion that the surface accessible to the solvent (Figure 1a) amounts to 1266 Å^2^, and the radius of gyration *R*_g_ (defined as the mass-weighted root-mean-square distance of the system atoms from their common c.o.m.) to 6.97 Å.

A somewhat different geometry is achieved in explicit water (Figure 1b), where a cubic cell with a size of 33.0 Å was adopted with 1091 water molecules and the MD run lasted for 500 ps, in view of the much faster relaxation due to the random collisions with the solvent. Here, the aCD assumes the typical truncated-cone shape taken by cyclodextrins in the solid state, but in the present case this feature is further enhanced by the clustering of the H chains in order to minimize the exposed surface. The PDF is again most useful to characterize the molecular shape induced by the environment. This feature can be seen in the PDF of the glycosidic oxygens of the macrocycle and of the S atoms plotted as before as a function of their distance from the macrocycle c.o.m. in Figure 2b, showing that the glycosidic oxygens at the secondary rim are much further from the c.o.m. that the S atoms carrying the H chains, which strongly cluster to minimize their contact with the water molecules. In this way, the macrocycle also achieves a large opening of the secondary rim so as to maximize the P-chain hydration.

**Figure 3:**
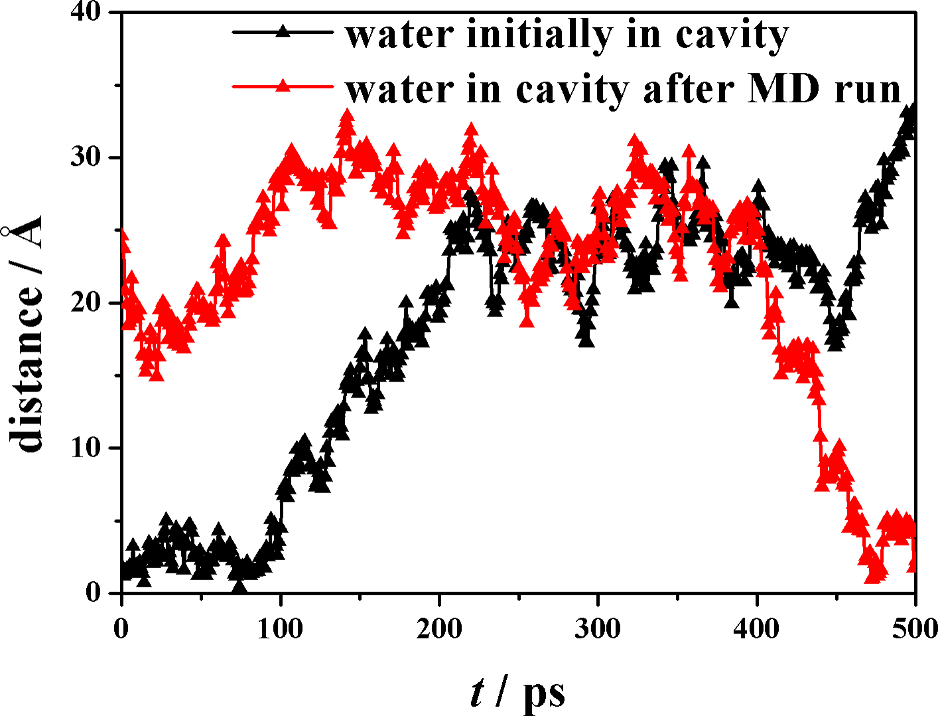
The distance between the oxygen atoms of two water molecules and the c.o.m. of the aCD plotted as a function of time calculated during the 500 ps MD run. One water molecule initially within the cavity escapes to bulk water (black symbols), being replaced by another one within the MD run (red symbols).

**Figure 4:**
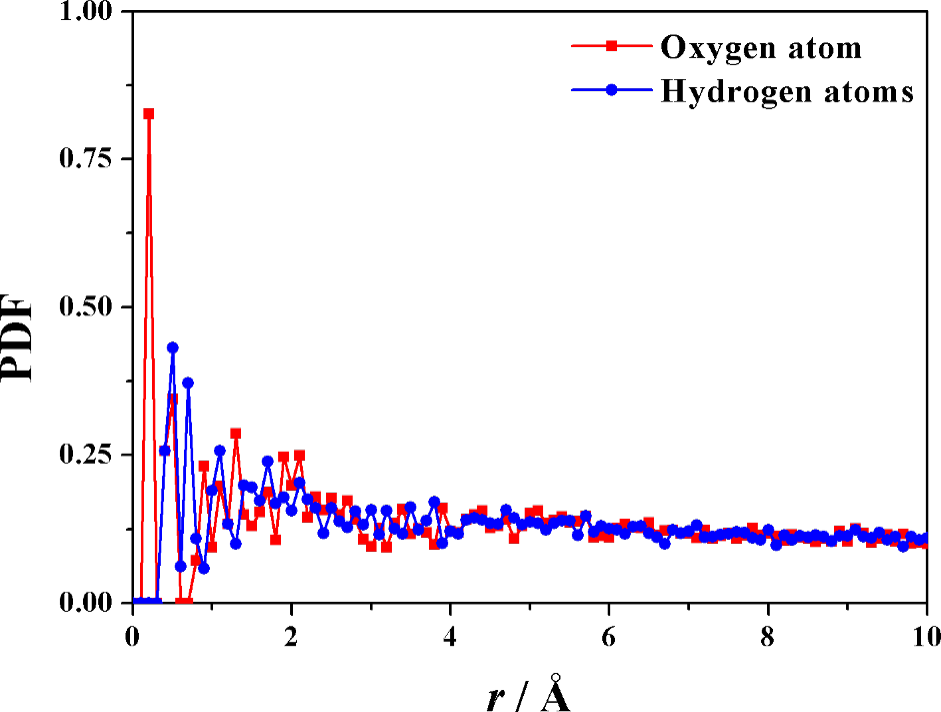
The PDF of the oxygen and of the hydrogen atoms of the water molecules (in red and in blue, respectively) plotted as a function of their distance from the aCD macrocycle c.o.m. calculated during the 500 ps MD run.

It should also be noted that there is a small cluster of five water molecules trapped into the cavity, and quite isolated from the bulk water, with a pattern rather similar to what found in the native β-CD [46]. Interestingly, also in this case there is a dynamic equilibrium involving the water molecules initially clustered within the hydrophobic CD cavity that are replaced by other molecules entering the cavity from the bulk water during the MD run. An example of this exchange process is shown by the trajectories (reported in Figure 3) of two water molecules in terms of the distance between their oxygen atoms and the c.o.m. of the hosting aCD plotted as a function of time: one of them is a water molecule entering the cavity (the water molecule evidenced in Figure 1b), while the other one is a water molecule initially comprised within the cavity that escapes to the outer bulk water. Moreover, the PDF of the atoms of the water molecules as a function of their distance from the macrocycle c.o.m. (see Figure 4) show that the cavity is populated throughout the MD runs, even though by different molecules.

In conclusion, in water the apolar H groups significantly cluster so as to minimize the contact with the environment, whereas the hydrophilic P groups show a marked opening to enhance their hydration. The ellipsoidal distortion of the macrocycle caused by the above mentioned interactions should also be noted. As a result, in water the surface accessible to the solvent is equal to 1358 Å^2^, while the radius of gyration *R*_g_ increases to 7.30 Å, with values significantly larger than what is obtained in vacuo (or in an apolar solvent).

### The interaction between two molecules

#### Simulations in vacuo

**Figure 5:**
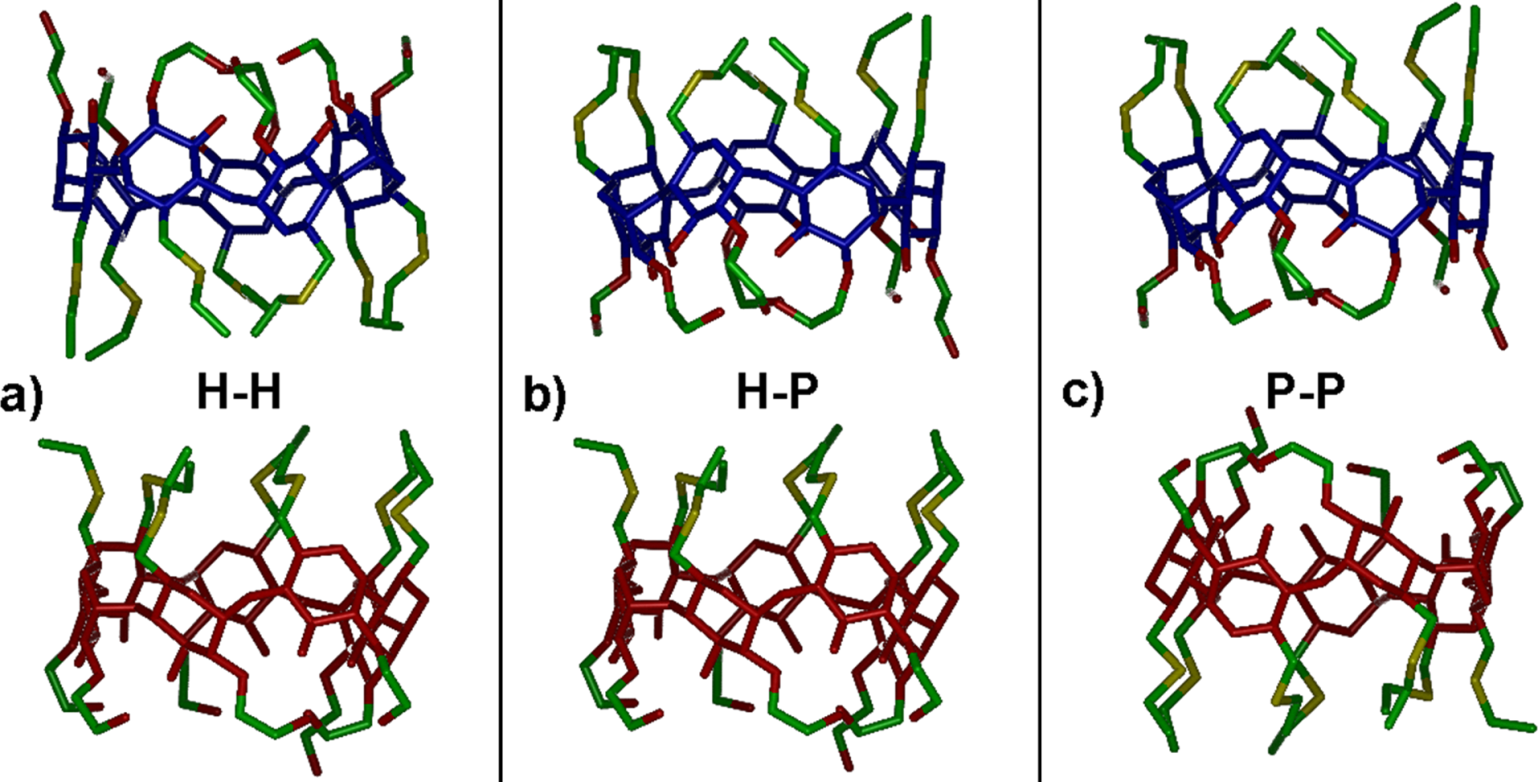
The pairwise initial arrangements of two amphiphilic molecules that face the two hydrophobic H groups, the hydrophobic H and the polar P groups, and the two polar P groups, from left to right in the order. The colour codes are as in Figure 1, while the macrocycles of the two aCD molecules are shown in blue and red for clarity.

The pairwise interaction between two amphiphilic CDs was investigated by facing two aCD molecules through their H groups, through one H and one P group, or through their P groups as shown in Figure 5, using the most stable optimized geometry obtained in vacuo.

**Figure 6:**
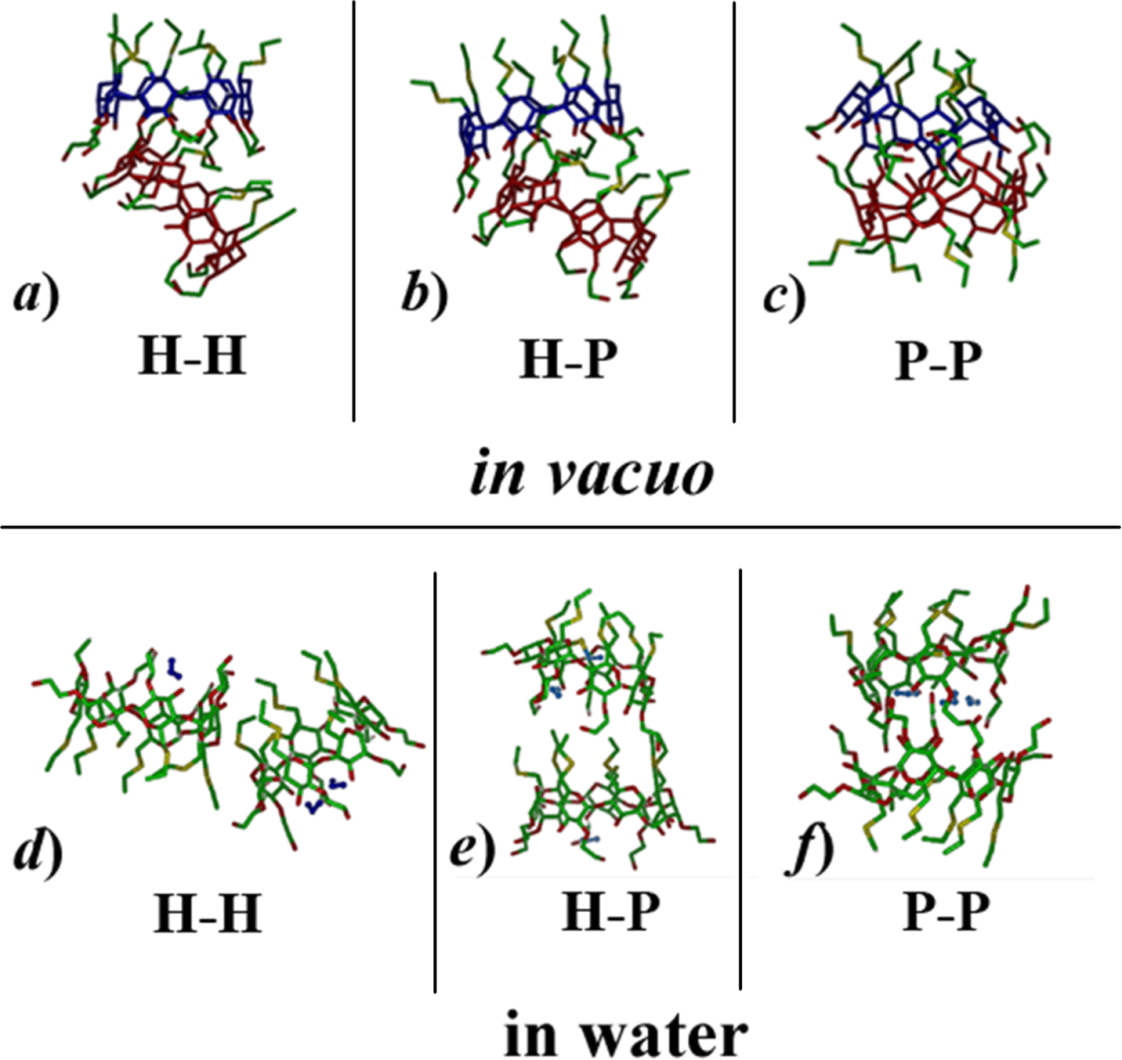
Final optimized geometries at equilibrium after the 30 ns MD runs obtained both in vacuo and in water. The Figure shows a line drawing of the dimeric aggregates (the hydrogen atoms were omitted for clarity). The atom colour codes are the same as in Figure 1.

The initial minimizations in vacuo yield a relatively weak interaction for the H–H arrangement involving the hydrophobic H groups through dispersive interactions, a stronger interaction in the P–P arrangement involving the polar P groups through mainly dipolar interactions and possible hydrogen bonds, and an even stronger interaction in the H–P arrangement, even though the additional stabilization only amounts to about 3 kJ/mol. It should be noted that while a P–P interaction may allow for intermolecular hydrogen bonds among the terminal OH groups, in the P–H arrangement a slightly larger number of intramolecular hydrogen bonds is actually present together with some shallow self-inclusion of two H groups. Moreover, the H–P arrangement does allow for a significant optimization of the dispersive interactions through partial inclusion of some H groups in the hydrophobic cavity of the other molecule. Significant changes are however achieved within the MD runs in vacuo lasting 30 ns, which allow for possible major rearrangements of the two molecules, as indeed found in the fully optimized geometries shown in Figure 6 at left. In particular, the H–H and H–P initial arrangements display an almost complete rotation and/or a noticeable tilt of one molecule with respect to the other one (Figure 6a and Figure 6b, respectively) leading in both cases to some favourable H–P interactions. The most stable geometry was found after the MD runs and final optimization of many instantaneous snapshots (100 snapshots taken at equilibrium in the final 10 ns when all the monitored quantities fluctuate around a constant average value) starting from the initial P–P geometry, which involves an interaction among the two polar groups. In this way, the two molecules can form seven intermolecular hydrogen bonds (in addition to the intramolecular ones) and optimize the dipolar interactions (Figure 6c) with the largest interaction energy, in absolute value, and the smallest radius of gyration but the largest surface accessible to the solvent (see Figure S1 of the Supporting Information File 1) as shown in Table 1. Here and in the following, the interaction energy is defined as *E*_int_ = *E*_aggr_ – *nE*_isol_, where *E*_aggr_ is the energy of the aggregate formed by *n* molecules and *E*_isol_ the energy of the isolated molecule. Interestingly, in this geometry the aggregate also shows a larger surface accessible to the solvent than in the other arrangements (see Table 1).

**Table 1:** Interaction energies.

Starting arrangement	In vacuo			In water		

	*E*_int_ (kJ/mol)	*R*_g_ (Å)	Accessible surface (Å^2^)	*E*_pot_^a^ (kJ/mol)	*R*_g_ (Å)	Accessible surface (Å^2^)

H–H	−251	8.17	1799	0	10.09	2333
H–P	−242	8.11	1771	54	9.74	2424
P–P	−266	7.94	1848	176	8.51	2112

^a^These are the average potential energies within the MD run with respect to the lowest one.

The two higher-energy geometries do not show major differences, since both have a favourable interaction of the P groups of one molecule with the H groups and with part of the lateral surface of the other molecule. Moreover, in either case there is inclusion of two H groups of one molecule in the hydrophobic cavity of the second one, and four intermolecular hydrogen bonds. In particular, the initial H–H arrangement yielded the final geometry of Figure 6a, with an interaction energy (see Table 1) intermediate between the most (Figure 6c) and the least stable one having the H–P arrangement (Figure 6b) due to somewhat weaker dipolar interactions of the latter one.

#### Simulations in water

The simulations in explicit water were carried out starting again from the initial arrangements shown in Figure 5 within a tetragonal cell with axes equal to 38 Å × 38 Å × 48 Å and 2086 water molecules. In water, the interaction between two amphiphilic β-CD is definitely weaker than in vacuo because of the competing interaction of the polar groups with the water molecules. In the initial minimizations, only minor changes were observed, mainly involving some clustering and partial shielding of the H groups to minimize contact with water. After the MD runs, only relatively loose aggregates were obtained, their optimized geometry being shown in Figure 6. The geometry of Figure 6d is the most stable one, as inferred by the potential energy averaged after equilibration within the final 350 ps of a preliminary dynamic trajectory lasting for 500 ps, while the geometries shown in Figure 6e and 6f have a higher average potential energy, as shown in Table 1. Further dynamic runs were carried out for a total of 30 ns to check for the robustness and stability of these geometries, but we did not detect any major change, neither in the potential energy, nor in the mutual arrangements of the two aCD (or more precisely in the distance between the centers of mass of the two aCD), which can require a longer simulation time to achieve equilibrium by small local rearrangements than potential energy. Accordingly, the initial interaction geometry kinetically traps the adducts in a deep local potential energy minimum, which may drive and affect the subsequent growth after addition of further molecules.

On the other hand, full optimization of the final snapshots produced as the minimum energy conformation the geometry of Figure 6e, even though the energy values of the optimized arrangements can be largely affected by the presence of the random, glassy arrangement of the water molecules trapped in some local energy minimum. The geometry of the aCD pair involves a weak interaction between a few P and H groups of the two molecules, producing a relatively open aggregate with a large surface accessible to the solvent shown in Supporting Information File 1, Figure S1 (see Table 1). Interestingly, the squared value of the radius of gyration is close to, though still smaller than, twice the squared radius of gyration of the isolated molecule in water, stressing again the relatively poor clustering of this dimeric aggregate. An analogous optimization for the other starting arrangements yielded the geometries shown in Figure 6d and Figure 6f. However, in the former case the aggregate has a quite large size, as shown by its radius of gyration, indicative again of a weak interaction with a quite large surface exposed to the water solvent, whereas in the latter case it has a significantly smaller radius of gyration and an even smaller exposed surface (see Table 1 and Supporting Information File 1, Figure S1). It must be pointed out that the size of the last arrangement could suggest stronger intermolecular interactions than in the previous cases mediated by the water molecules entrapped by the P groups, but this arrangement is not the most stable one in view of its higher energy, related in turn with the presence of H groups exposed to water.

### The interaction among four molecules

#### Simulations in vacuo

**Figure 7:**
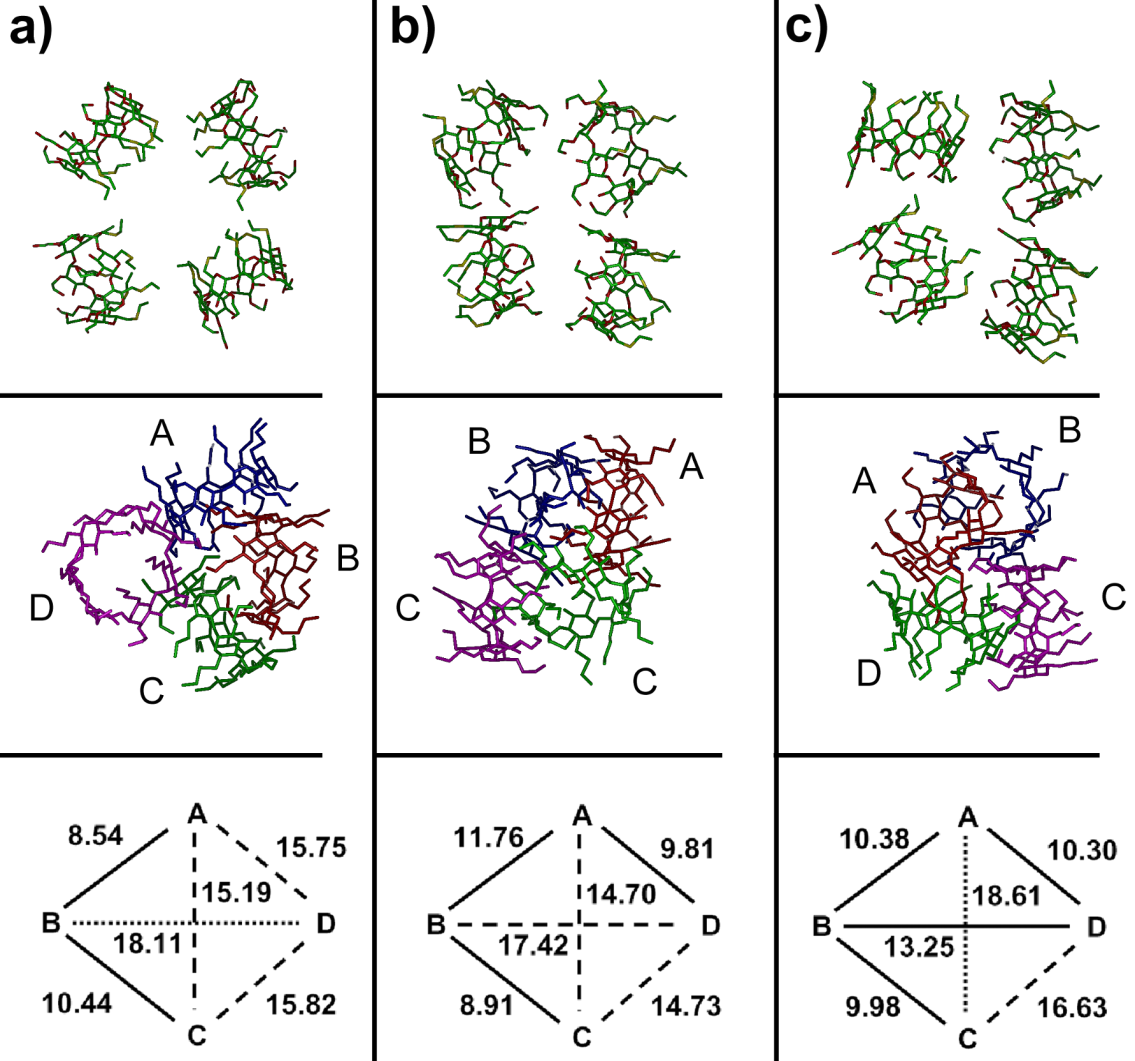
The starting arrangements (a–c) of the four α-CD molecules (top row) and the final arrangement after the MD run and the final optimization in vacuo (second row). The bottom row shows a schematic pattern of the distances (in Å) between the centres of mass of the four macrocycles averaged over the last 10 ns of the MD run (out of a total of 30 ns). The solid lines indicate the short distances (<14 Å), the dashed lines the longer distances (in the range between 14 and 18 Å) and the dotted lines the still larger separations (>18 Å). In general the standard errors of the mean are ≤0.01 Å, while the standard deviation around the mean are in the range ±(0.3–0.5) Å.

The stability of larger aggregates was then investigated considering four molecules interacting in different relative orientations. In the starting arrangements, the four molecules can interact through a) the four H groups, b) the four P groups, or c) two P groups, one H group and a side surface, thus being essentially placed at random (first row of Figure 7). The initial minimizations already produced significant interactions among the molecules, which approached one another with only minor changes. The interactions energies turned out to be quite significant, and increasingly larger in the above-mentioned order, with weaker interactions among the H groups only due to dispersion forces in case a, and stronger interactions in cases b and c due to the presence of intermolecular hydrogen bonds and dipolar interactions.

The subsequent MD runs of these geometries, each lasting for 30 ns, led in some cases to significant rearrangements, always producing single aggregates where the four molecules are kept together by different combinations of dispersion forces, dipolar interactions and hydrogen bonds. However, there are small but significant differences in the aggregation pattern, as it is already evident from simple inspection of the final optimized geometries of Figure 7. The most stable state arrived in cases a and b of Figure 7 approximately shows the same stability in vacuo, as shown by their interaction energies reported in Table 2. However, the aggregation patterns are very different, with important implications for the interactions of larger clusters. In fact, in the case of Figure 7a the MD run leads to large rearrangements such that one molecule (molecule B in Figure 7) undergoes a complete rotation in order to optimize its intermolecular interactions through inclusion of two of its P groups in the hydrophobic cavity of two neighbouring molecules (molecules A and C), thus acting as a bridge between them, showing also self-inclusion of one of its H groups. As a result, three molecules are quite close to one another, whereas the fourth one (molecule D) is farther away, being connected more loosely to the other ones through a few intermolecular hydrogen bonds.

To better classify these aggregates, let us conveniently denote as closer molecules those showing a distance d between their c.o.m. smaller than 14 Å, i.e., roughly twice the value of the radius of gyration of the isolated molecule, and farther molecules those with a larger *d*. These distances are graphically shown in the fourth row of Figure 7, where the thick lines denote the separation between the closer molecules (*d* < 14 Å), the dashed lines the slightly longer distances (14 Å < *d* < 18 Å), and the dotted lines the farther molecules (*d* > 18 Å). In case of Figure 7a, molecule D is somewhat farther away, as implied by the *d* values involving it. Accordingly, we may denote this as a 3 + 1 aggregate, and in fact the whole system has a relatively large *R*_g_ value and a large surface accessible to the solvent (see Table 2 and Supporting Information File 1, Figure S2).

**Table 2:** Solvent-accessible surface.

Starting arrangement^a^	In vacuo		

	*E*_int_ (kJ/mol)	*R*_g_ (Å)	Accessible surface (Å^2^)

a	−678	11.26	3541
b	−679	10.59	3139
c	−731	11.05	3381

^a^The three arrangements are labelled as indicated in Figure 7.

The case of Figure 7b has about the same stability, as said before, due to a different combination of dispersion interactions and hydrogen bonds. In this case, in fact, molecule D shows both self-inclusion of a P group and inclusion of another, adjacent P group in the cavity of the neighbouring molecule A. Moreover, molecule B includes one of its P groups in the cavity of molecule C, forming also a hydrogen bond with a glycosidic oxygen of the latter macrocycle. Accordingly, this aggregate could be identified as a tight 2 + 2 cluster held together by intermolecular hydrogen bonds between two pairs of molecules, but it may also be denoted as a veritable 4 cluster in view of the small value of the radius of gyration (10.59 Å, see Table 2) and of the distances *d* shown in Figure 7 showing that all the molecules are quite close together. Correspondingly, in this arrangement the surface exposed to the solvent is also quite small (see again Table 2 and Supporting Information File 1, Figure S2). Finally, case 7c shows the most stable aggregate with the largest *E*_int_, in absolute value (see Table 2) due to strong interactions with mutual inclusion of H and P groups in neighbouring macrocycles. Thus, in addition to a shallow self-inclusion of an H group, molecule B of Figure 7c shows inclusion of one H group in the macrocycle of C, and of two H groups in the macrocycle of A. Moreover, molecule A shows inclusion of one P group within the macrocycle of D. Thanks also to the intermolecular hydrogen bonds, involving molecules A and D, and molecules B and C, the aggregation leads to rather short distances among the c.o.m. of the closer molecules, as shown in the last row of Figure 7, so that this is again a 4 cluster. On the other hand, this inclusion pattern leads a more “open” aggregate, in view of the quite long A–C separation, producing a quite large radius of gyration and a relatively large exposed surface, as shown in Table 2 and Supporting Information File 1, Figure S2. As a conclusion of this paragraph, we point out that if the *E*_int_ values for the aggregates of four molecules are normalized by the number of interacting molecules, we get quite larger values (in absolute value) than for two molecules, though not by a factor of six (the number of pairwise interactions among four molecules). Such a result suggests cooperative effects favouring larger clusters compared to smaller ones, even though the four molecules cannot simultaneously optimize all the possible pairwise interactions for steric reasons.

#### Simulations in water

**Figure 8:**
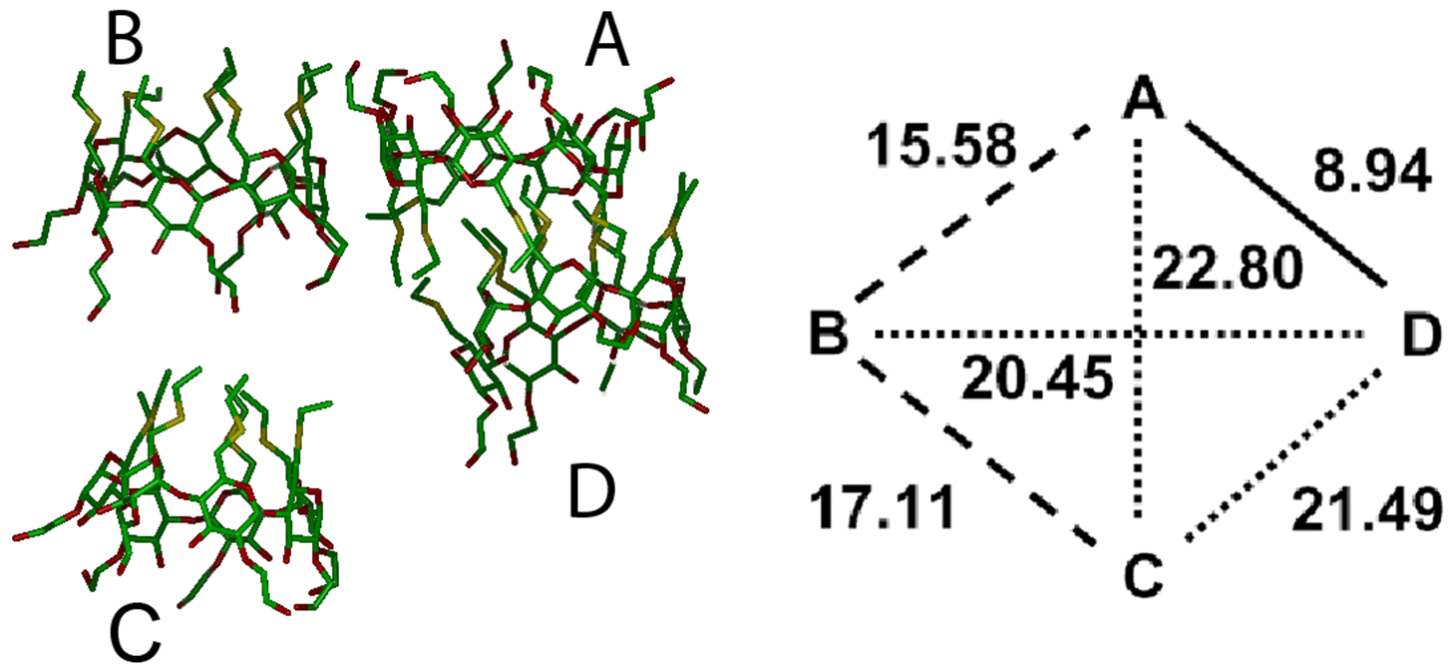
The optimized geometry achieved by four aCD molecules in water by four molecules after the MD run. The line drawing of the aggregate (at left) and a schematic pattern of the distances (in Å, at right) between the centres of mass of the four macrocycles is shown (see Figure 7 for more details).

The simulations in water of larger systems of aCD in water are computationally more demanding, and accordingly here we only report our preliminary results, already providing interesting information, deferring to a future paper a more detailed analysis. The simulation of four aCD in water was carried out in a large cubic cell with an axis equal to 60 Å and 6806 water molecules starting with the arrangement shown in Figure 7a where the H groups point towards the common centre of mass. This arrangement somehow shields the hydrophobic chains from water while exposing the polar chains to water, but since we obtained rather soon an interaction pattern similar to what obtained in vacuo with four molecules and also in water with eight molecules, we did not consider the other arrangements of Figure 7 for brevity. In fact, the final, optimized geometry achieved in water after an MD run of 1 ns, shows a strong interaction between two molecules (molecules A and D in Figure 8), as shown by the the short distance between the centers of mass of their macrocycles. These molecules are somewhat off-axis so as to optimize the interactions between their H groups that tightly inter-digitate, with a mutual shallow inclusion of a few of them into the cavity of the facing molecule. Furthermore, there is a looser side interaction of a third molecule (molecule B in Figure 8), interacting with the A and D molecules through dispersion interactions involving a few H groups of the B molecule and the P groups of the A molecule. An even looser interaction with these molecules is shown by the fourth one (molecule C in Figure 8), which anyway is sufficient to keep it aligned with molecule B along an axis passing through average planes formed by the CD macrocycles. The weakness of this interaction can also be gauged by the conformation of the latter molecule that closely matches the shape of the isolated molecule in water, both for the tight clustering of the H groups to minimize the hydrated surface and for the wide opening of the P groups to maximize their hydration. In conclusion, even though one could denote this arrangement as a 2 + 1 + 1 aggregate, it is best described as a 3 + 1 aggregate. There is a further observation supporting this conclusion. In fact, the radius of gyration of the whole cluster is much larger than in vacuo, amounting to 13.43 Å. On the other hand, the cluster formed by the closer molecules (A, B and D in Figure 5) has a radius of gyration of 11.71 Å, but the surface exposed to the solvent is quite small (see Supporting Information File 1, Figure S3), amounting to 3154 Å^2^. Even though the size of this cluster is still larger than the value obtained in vacuo for the whole aggregate of four molecules, it favourably compares with the values of the two clusters of three molecules achieved in water with a larger system, as described in the next section.

### The embryonic micelle: random aggregation of eight molecules

#### Simulations in vacuo

**Figure 9:**
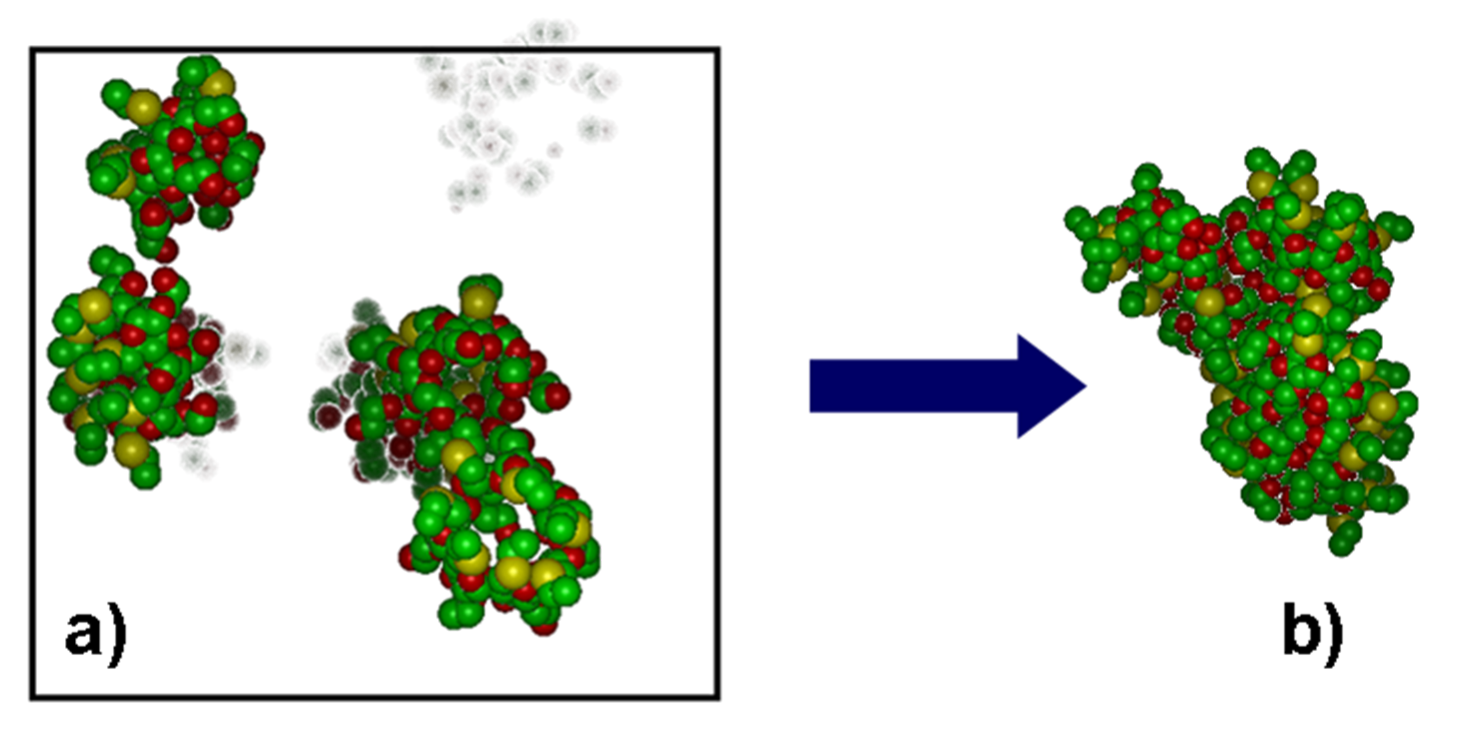
a) The initial random arrangement of eight molecules of the model aCD in a space-filling representation within the simulation box (note that the overlap of the molecules is only apparent). b) The optimized geometry of the aggregate formed by eight molecules of the model aCD in vacuo. The hydrogen atoms were omitted for clarity, while the atoms colour code is the same as in the line drawings of Figure 1.

In order to better investigate the early stage of the nucleation of larger aggregates or possibly veritable micelles formed by the amphiphilic CDs, we first investigated the association behaviour of eight molecules in vacuo to model an apolar, weakly interacting solvent. To this purpose, we randomly placed the molecules with an unbiased arrangement in a cubic cell with a size of 61.5 Å using periodic boundary conditions (Figure 9a).

The initial minimization already yielded a very large, but relatively loose aggregate. In the subsequent MD run lasting for 15 ns, such an aggregate turned out to be quite stable, further enhancing the intermolecular interactions. The final, optimized geometry is shown in Figure 9b: the eight molecules do strongly interact both through the intermolecular hydrogen bonds and through mutual inclusion of the side chains in the cavity of adjacent molecules, basically repeating on a larger scale the interaction pattern of smaller aggregates. Also, the radius of gyration of the whole aggregate has a relatively small value *R*_g_ = 13.55 Å. Interestingly, this value is slightly less than twice the value of the single molecule, 6.96 Å, and since the volume pervaded by a molecule or an aggregate scales as *R*_g_^3^, it turns out that the volume of the aggregate is somewhat less than eight times the volume of the single molecule thanks to the attractive intermolecular interactions.

#### Simulations in water

**Figure 10:**
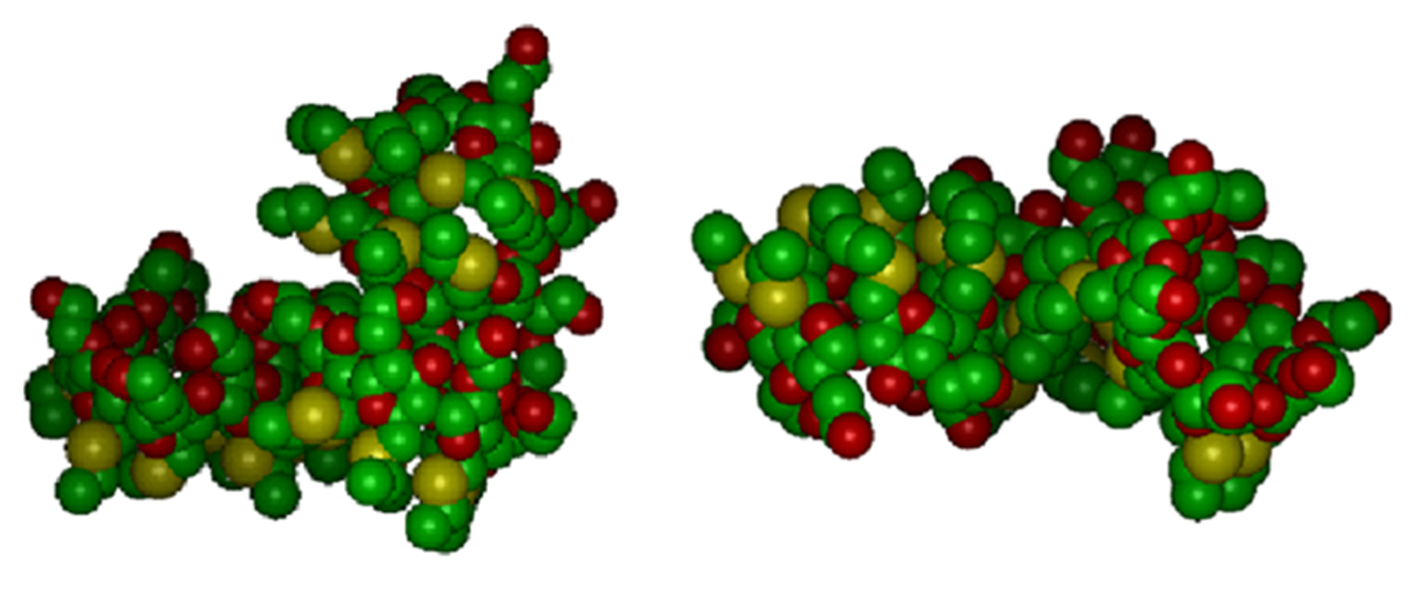
The two aggregates obtained in water, each comprising three molecules of the model aCD, cluster A (at left) and cluster B (at right) in a space-filling representation. The hydrogen atoms were omitted for clarity, while the atoms colour code is the same as in the line drawings of Figure 1.

The simulations in explicit water adopted the same starting arrangement as in vacuo into the same periodic cell, which required the presence of 6250 solvent molecules to achieve the bulk water density. In water, the initial minimization led to a very poor clustering of a few molecules, not yet corresponding to a real aggregate. The subsequent MD runs produced some rearrangements which could thus form veritable, although still loose aggregates, which however did not show any tendency to coalesce into larger ones. After 2 ns of simulation time, the system appeared to have achieved a (pseudo) equilibrium state, as monitored through the system energy and intermolecular distances within each formed aggregate. In this case, the whole system comprised two aggregates, each formed by three molecules and denoted in the following as clusters A and B (see Figure 10), quite similar to the aggregate formed by molecules A, B, D in Figure 8, together with two isolated molecules.

**Figure 11:**
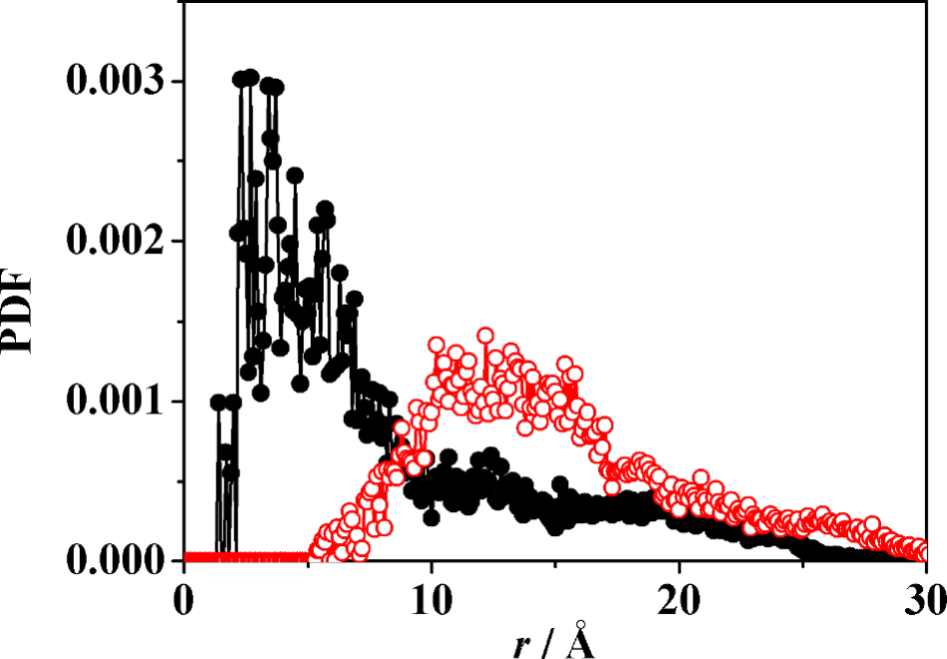
The PDF of the S atoms of the H groups at the primary rim (black symbols) and of the oxygen atoms of the hydroxyl groups at the secondary rim and in the P groups (red symbols) in cluster A plotted as a function of their distance *r* from the cluster c.o.m.

Cluster A presents inclusion of a P group of one molecule in the cavity of a second, neighbouring molecule, while the third one interacts with the latter through dipolar and dispersive interactions at their lateral surfaces. It is interesting to note that in this cluster the S atoms of the H groups tend to be close to the c.o.m. of the aggregate during the MD run, as shown by the PDF of Figure 11. Moreover, the P groups, and in particular the secondary hydroxyl groups of the macrocycles and the terminal ones of the P groups tend to stay in the outer region to enhance the overall hydration. On the other hand, no inclusion is present in cluster B, where the three molecules are held together by dipolar and dispersion interactions taking only place at their outer surfaces. Note that this nanostructure could be viewed as the building block of a vesicle surface [37]. In any case, the radius of gyration of the two clusters are essentially equal, since they amount to 12.01 Å and 12.09 Å, respectively, showing again the relatively loose association achieved in water in this stage. It should be stressed, however, that these values are only marginally larger than the value of 11.71 Å achieved in water for the aggregate of the three closer molecules discussed in the previous section. This result suggests that this cluster size is indeed quite favourable in this initial pseudo equilibrium aggregation stage that may persist for quite a long time.

**Figure 12:**
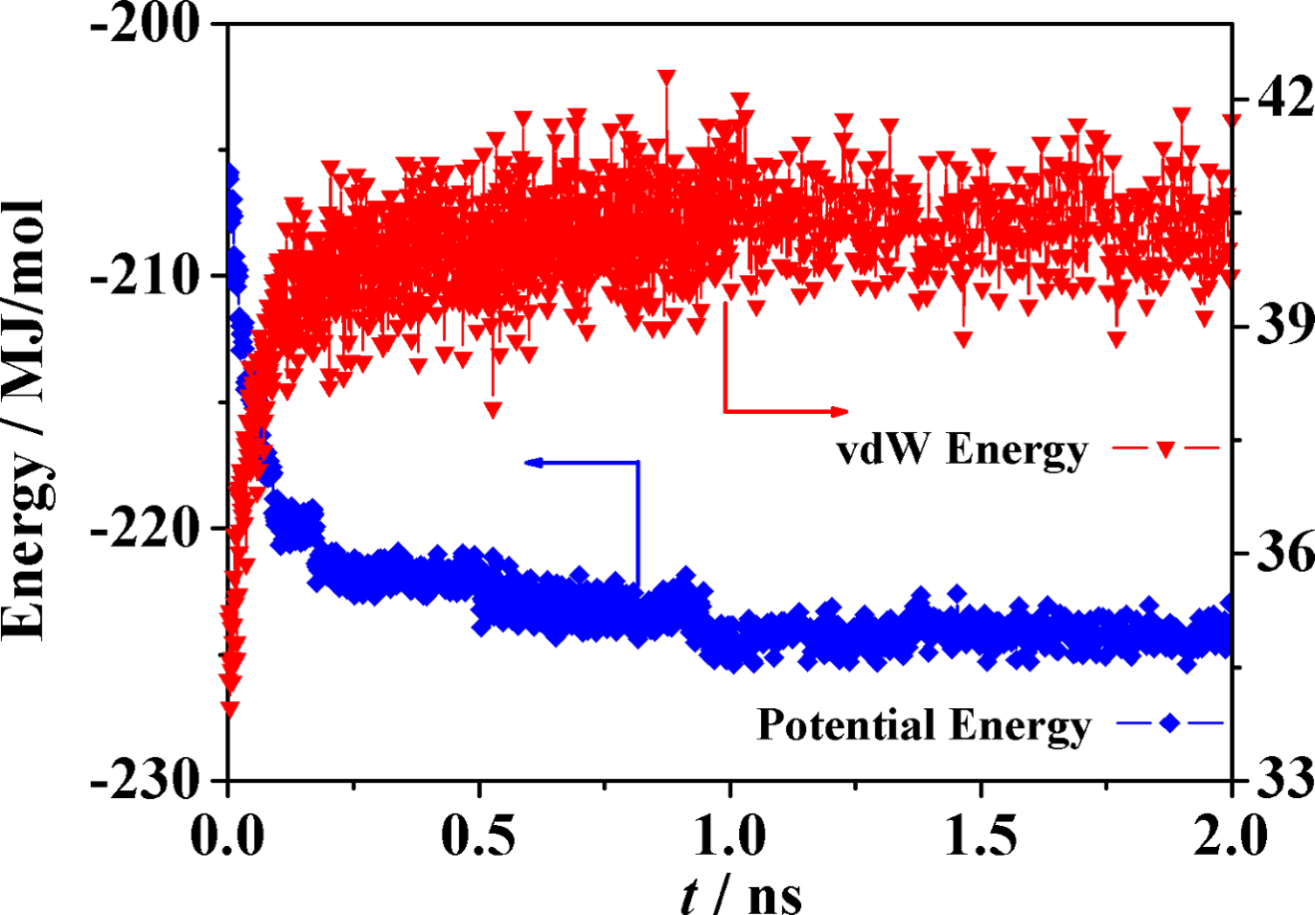
The time change of the potential energy and of the van der Waals energy due to the dispersion and covolume interactions in the MD run of eight molecules of the model aCD in water, showing the apparent equilibration after about 1 ns.

The aggregation process led to an apparent equilibration, as suggested inter alia by the lack of change in the potential and van der Waals energy of the whole system in the last half of the MD run (see Figure 12). Of course much lengthier processes cannot be ruled out: in fact, in view of the small size of these aggregates and of the simulations carried out in vacuo with four and eight molecules, the present results only describe the embryonic stage of aggregation, separated from later stages by some free energy barrier, mainly due to configurational entropy. On the other hand, taken together the present results in water may provide some clues about the possible kinetics of aggregation: at first there is the fast formation of small clusters comprising few molecules, followed by the further aggregation of these cluster with may add individual molecules but also coalesce more slowly because of their smaller diffusivity related in turn with their larger size.

**Figure 13:**
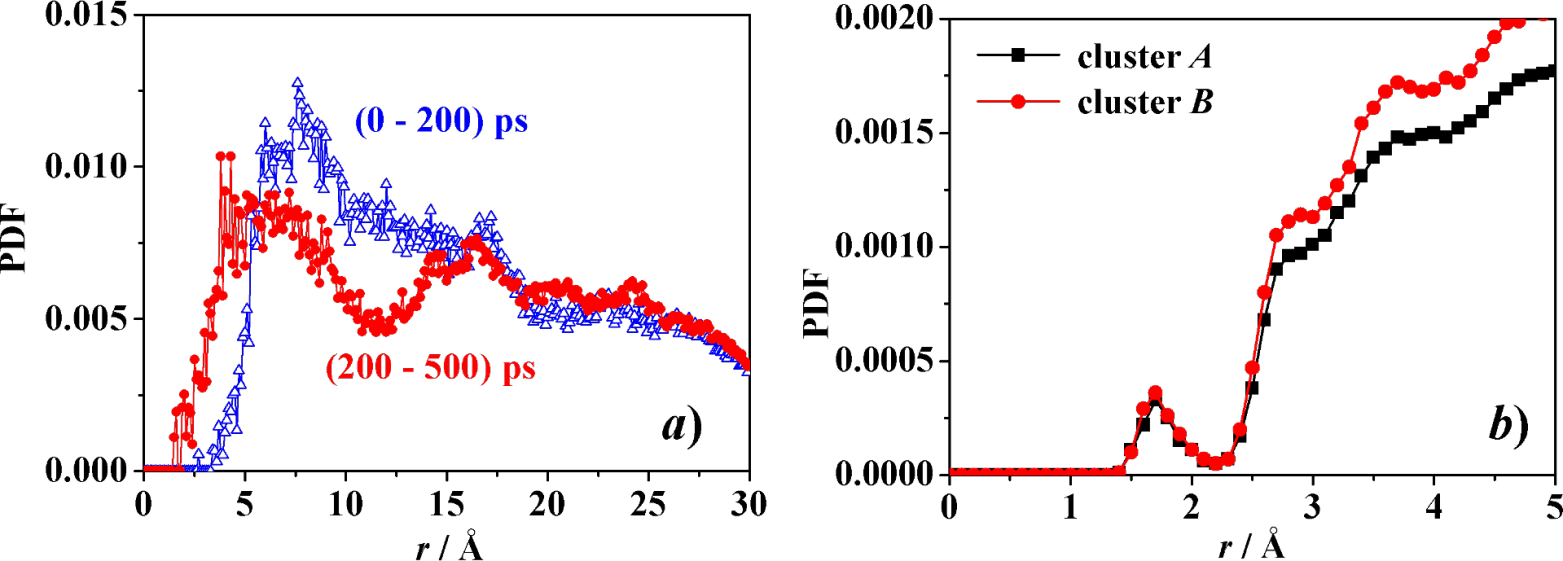
a) The PDF of the eight molecules of the model aCD in water as a function of their distance *r* from the common c.o.m. b) The pair distribution function of the water molecules (more precisely, of their oxygen atoms) as a function of their distance from the atoms belonging to each of the two clusters of three molecules (see Figure 10).

The MD run in water yielded also an increase of the intermolecular order, as shown by the change in the pair distribution function PDF of Figure 13a within the initial part of the MD run. In particular, in the PDF the first peak centred at about 8 Å from the common c.o.m. within the initial 200 ps of the MD run suggests that a few molecules cluster near it, while other farther molecules lead to a broad distribution of distances roughly centred at 18 Å, indicating also a large and independent molecular mobility. Later on, within the following 300 ps the PDF shows a broad first peak at about 6–7 Å from the common centre of mass, followed by a second well-defined peak at about 17 Å and then a broad shoulder at larger distances. These features suggest the embryonic formation of a more structured system corresponding to the formation of clusters A and B.

As for the system hydration, it can be described through the PDF of the water molecules (or equivalently of their oxygen atoms) as a function of their distance *r* from the atoms of the two clusters, shown in Figure 13b. The first peak at about *r* = 1.7 Å is due to the O–H^…^O_w_ hydrogen bonds of the hydroxy groups of the cluster with the water oxygens (indicated as O_w_), while the two peaks or shoulders at about *r* = 2.9 Å and 3.7 Å are mainly due to the O^…^O_w_ non-bonded distances of the two first hydration shells. Note also the slightly larger value of the PDF for the cluster B due to its more “open” shape that produced an effectively larger accessible surface for the solvent.

## Conclusion

The supramolecular aggregation of molecules is an important phenomenon determined both by their interactions in a specific environment (hence on their concentration and on the solvent) and by their shape, which may preferentially determine stable mesophases, ranging from micelles to membranes, or even liquid crystals. It is well-known that the shape and the interactions among native or modified cyclodextrins can drive specific packing in the solid state, but also in solutions these factors are crucial in driving the nucleation and then the large-scale aggregation, inducing the observed arrangements of micelles and/or vesicles and/or nanospheres. It is in general very difficult to model and understand at the atomistic level these events, but theoretical studies based on molecular mechanics and molecular dynamics simulations can yield a most useful “bottom up” approach to model amphiphilic cyclodextrins that may interact in vacuo or in water.

The simulation results reported in the present paper show that non-ionic amphiphilic β-CD (aCD) carrying short hydrophobic (thioethyl) and polar (ethylene oxide) substituents at opposite rims can aggregate with a relatively complex interaction pattern. In fact, the hydrophobic H groups and the polar P groups may compete for either self or mutual inclusion in their own or in a neighbouring hydrophobic cavity. Such patterns were monitored by MD simulations in vacuo and in water, which suggest that all these interactions are present, at least in the embryonic aggregation stage, while the expulsion of a few water molecules clustered within the hydrophobic cavities of the aCD entropically favours the process. Interestingly, the simulations in explicit water suggest the clusters of three molecules of the model aCD are quite robust, and may coexist with isolated molecules for a while (at least for nanoseconds, according to the present preliminary simulations), whereas the simulations in vacuo suggest the relative fast formation of larger aggregates comprising all the molecules included in the simulations. While specific solvation effects cannot be ruled out, we point out that in vacuo all kinetic processes are much faster than in explicit water because of the lack of the solvent viscosity (the random collisions with the water molecules). Accordingly, the results obtained in vacuo suggest that larger aggregates might eventually form in water as well, possibly with unlike arrangements, so that the present results give a picture of the early nucleation stage of the larger aggregates that are experimentally observed. Note also in this context that our results suggest also the presence of robust, though metastable arrangements that may persist also after addition of further aCD molecules, with similar but unlike interaction geometries.

It should be noted that a qualitatively similar pattern was indeed experimentally observed in particular by light scattering studies [24]. In fact, the observed scattered intensity obtained for an aCD similar to compound **1** of Scheme 1 but with a longer polar chain with *n* = 1 (on the average), could be well interpreted as due to the diffusive behaviour of isolated molecules, of small micelles of a few molecules and of much larger aggregates that coexist at equilibrium, even though no quantitative comparison can be made with the present results obtained for a model compound. It should be added that in the same paper [24] the presence of the small micelles and of the larger aggregates was independently confirmed by small-angle X-ray and dynamic light scattering experiments, respectively. We further note that aCD with longer alkyl chains (compound **2** of Scheme 1, data not shown) did not show the presence of isolated molecules in equilibrium with small micelles and larger clusters [24]. In this case, the aCD would show a relatively more hydrophobic nature than our model aCD. We can thus speculate that mutual inclusion of the polar chains in the cavity of neighbouring molecules as found in the present paper would be more unlikely because of the enhanced hydrophobic interactions among the alkyl chains: their cooperative effect would then favour the micelle formation *vs.* the isolated molecules in spite of the entropy loss entailed by the clustering process.

As a final remark, we point out that with atomistic MM and MD methods we can model the first nucleation steps which may take place both in apolar solvent and in water in terms of the geometry of the aggregates and of their interaction energy in a given solvent. In the proposed approach the different shapes assumed by aCD and the non-covalent interactions with the solvent may lead to different macro-aggregates, either micelles or bilayer, or vesicles and nanospheres at appropriate concentrations. In the recent past, some of these structures have been selected to yield versatile and reliable carriers for drug delivery [3,50], and even for molecular recognition of polymers [51]. In this scenario, the proposed study can open new perspectives in the design of aggregates and correlate their structures with the physico-chemical properties.

## Supporting Information

File 1 Pictures of the surface accessible to the solvent for the aggregates of two and four aCD molecules.
