# Supplementary material for: Aggregation behaviour of amphiphilic cyclodextrins: the nucleation stage by atomistic molecular dynamics simulations
Source: Beilstein J Org Chem. 2015 Dec 7;11:2459–73. doi: 10.3762/bjoc.11.267 (PMC4685891; doi:10.3762/bjoc.11.267)
Supplement: File 1 — Pictures of the surface accessible to the solvent for the aggregates of two and four aCD molecules. [file Beilstein_J_Org_Chem-11-2459-s001.pdf]

Supporting Information

for

Aggregation behaviour of amphiphilic cyclodextrins: the nucleation stage by atomistic molecular dynamics simulations

Giuseppina Raffaini[*1,2,§] Antonino Mazzaglia[3] and Fabio Ganazzoli[1,2]

Address: [1]Dipartimento di Chimica, Materiali e Ingegneria Chimica ‘G. Natta’, Politecnico di Milano, via L. Mancinelli 7, 20131 Milano, Italy, [2]Unità Politecnico, INSTM, piazza Leonardo da Vinci 32, 20133 Milano, Italy and [3]CNR-ISMN Istituto per lo Studio dei Materiali Nanostrutturati, c/o Dipartimento di Scienze Chimiche, Biologiche, Farmaceutiche ed Ambientali dell’Università di Messina, Via F. Stagno d'Alcontres 31, 98166 Messina, Italy

Email: Giuseppina Raffaini - giuseppina.raffaini@polimi.it

*Corresponding author

[§]Tel. +39-0223993068; Fax +39-0223993081

Pictures of the surface accessible to the solvent for the aggregates of two and four aCD molecules

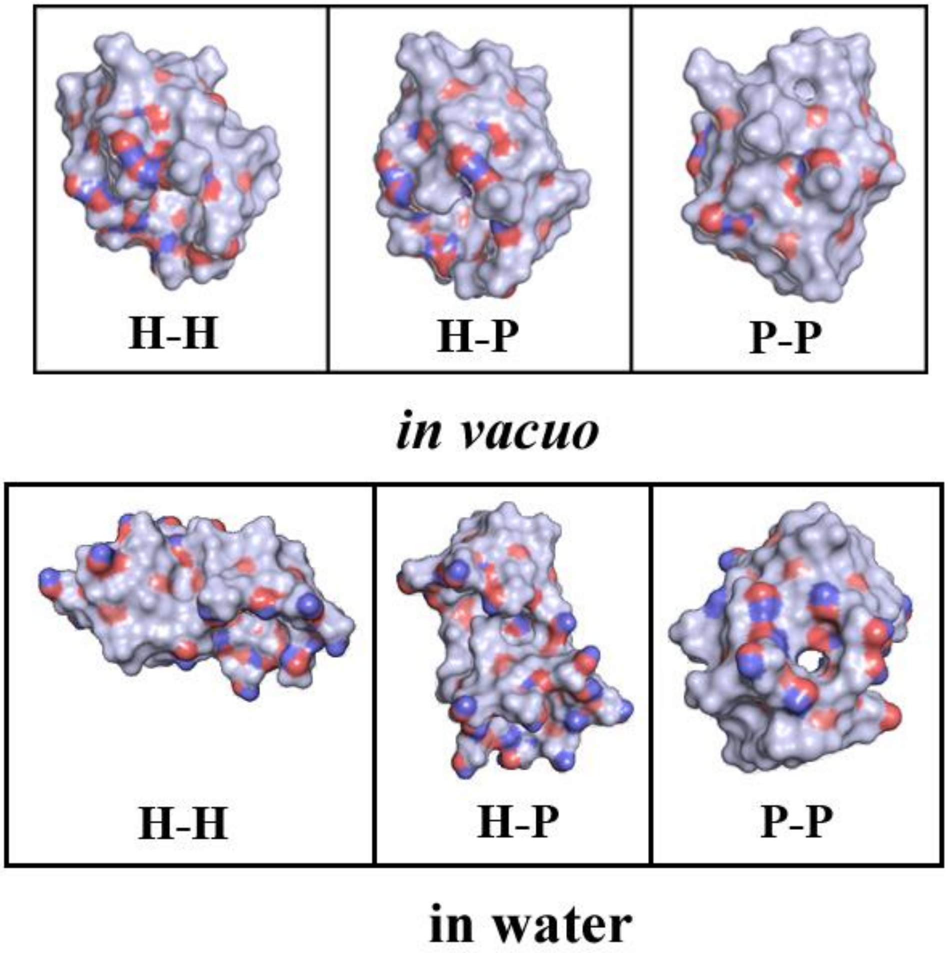

Figure S1: The surface accessible to the solvent, considered as a spherical probe with the radius of 1.4 Å, for the aggregates of two molecules (the electrically neutral part of the molecule is in grey, the negative areas of the oxygens are in red and the positive ones of the hydroxyl hydrogens in blue as in Figure 1).

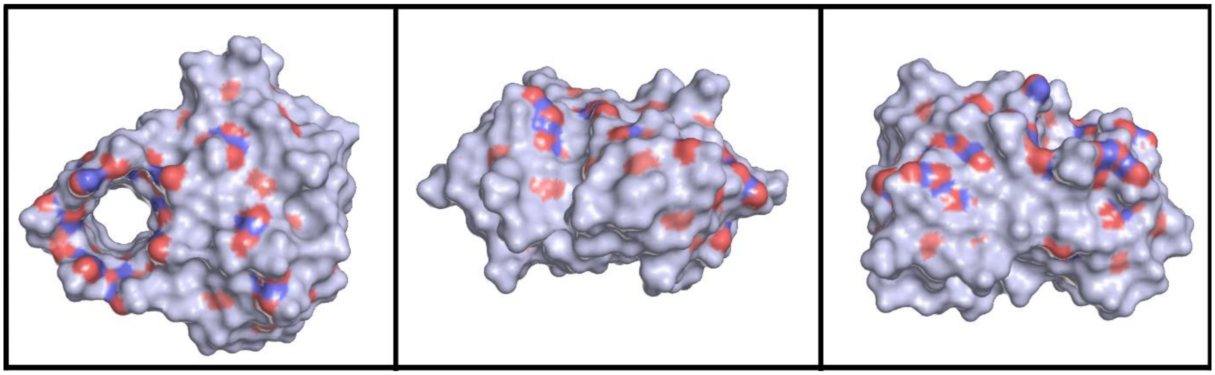

Figure S2: The surface accessible to the solvent for the aggregate of four molecules in vacuo (the colour code is the same as in Figure S1 and in Figure 1). Note the hydrophobic side in the lower part of the aggregates (grey colour) and the polar side in the upper part (red and blue colours).

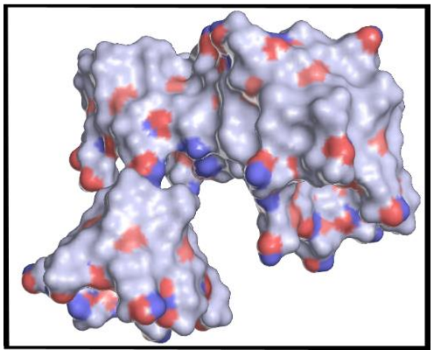

Figure S3: The surface accessible to the solvent for the aggregate of four molecules in water (the colour code is the same as in Figure S1 and in Figure 1).
